# A high utility integrated map of the pig genome

**DOI:** 10.1186/gb-2007-8-7-r139

**Published:** 2007-07-11

**Authors:** Sean J Humphray, Carol E Scott, Richard Clark, Brandy Marron, Clare Bender, Nick Camm, Jayne Davis, Andrew Jenks, Angela Noon, Manish Patel, Harminder Sehra, Fengtang Yang, Margarita B Rogatcheva, Denis Milan, Patrick Chardon, Gary Rohrer, Dan Nonneman, Pieter de Jong, Stacey N Meyers, Alan Archibald, Jonathan E Beever, Lawrence B Schook, Jane Rogers

**Affiliations:** 1 The Wellcome Trust Sanger Institute, Wellcome Trust Genome Campus, Hinxton, Cambridge CB10 1SA UK; 2 College of Agriculture, Consumer and Environmental Sciences, University of Illinois at Urbana-Champaign, Urbana, Illinois 61801 USA; 3 Laboratoire de Génétique Cellulaire, INRA, 31326 Castanet-Tolosan, France; 4 INRA-CEA, Domaine de Vilvert, 78352, Jouy en Josas cedex, France; 5 US Department of Agriculture, Agricultural Research Service, US Meat Animal Research Center, Clay Center, NE 68933-0166, USA; 6 Children's Hospital Oakland-BACPAC Resources, Oakland, California 94609, USA; 7 Roslin Institute, Roslin, Midlothian EH25 9PS, UK

## Abstract

**Background:**

The domestic pig is being increasingly exploited as a system for modeling human disease. It also has substantial economic importance for meat-based protein production. Physical clone maps have underpinned large-scale genomic sequencing and enabled focused cloning efforts for many genomes. Comparative genetic maps indicate that there is more structural similarity between pig and human than, for example, mouse and human, and we have used this close relationship between human and pig as a way of facilitating map construction.

**Results:**

Here we report the construction of the most highly continuous bacterial artificial chromosome (BAC) map of any mammalian genome, for the pig (*Sus scrofa domestica*) genome. The map provides a template for the generation and assembly of high-quality anchored sequence across the genome. The physical map integrates previous landmark maps with restriction fingerprints and BAC end sequences from over 260,000 BACs derived from 4 BAC libraries and takes advantage of alignments to the human genome to improve the continuity and local ordering of the clone contigs. We estimate that over 98% of the euchromatin of the 18 pig autosomes and the X chromosome along with localized coverage on Y is represented in 172 contigs, with chromosome 13 (218 Mb) represented by a single contig. The map is accessible through pre-Ensembl, where links to marker and sequence data can be found.

**Conclusion:**

The map will enable immediate electronic positional cloning of genes, benefiting the pig research community and further facilitating use of the pig as an alternative animal model for human disease. The clone map and BAC end sequence data can also help to support the assembly of maps and genome sequences of other artiodactyls.

## Abstract 2

A new physical map of the bovine genome has been constructed by integrating data from genetic and radiation hybrid maps, and a new bovine BAC map, with the bovine genome draft assembly.

## Background

The pig is a domesticated eutherian mammal and a member of the Cetartiodactyla order, a clade distinct from rodent and primates that last shared a common ancestor with man between 79 and 87 million years ago [1]. It has co-evolved with humans for several thousand years and today has considerable economic importance as a source of meat-based protein. The pig is also being used increasingly in biomedical research for studies of a spectrum of human diseases that may be modeled less well in rodents, including obesity, arthritis, cardiovascular disease, and skin and eye conditions [2,3]. An area of particular interest has been its potential to supply organs, tissues and cells for transplant through so called xenotransplantation (that is, transplantation of pig organs to humans), providing that issues surrounding porcine endogenous retroviruses, cytomegaloviruses and lymphotropic herpesvirus can be resolved [4,5].

To promote the identification of genes associated with single gene disorders and the mapping of quantitative trait loci (QTL), a variety of genomic resources have been developed in recent years [6]. Microsatellite (ms) linkage maps and whole-genome radiation hybrid (RH) maps have been constructed that contain over 3,000 markers and provide resolution down to approximately 1 Mb [7-10].

Several large insert bacterial artificial chromosome (BAC) clone libraries have been generated [11,12]. The libraries have been used successfully to isolate and characterize pig genes and to map and sequence small regions of the genome of particular interest, for example the major histocompatibility complex [13], and the killer immunoglobulin-like receptor gene [14]. The characterization of specific genes has been facilitated by expressed sequence tag sequencing projects that have generated approximately one million sequences in the public domain. A low genome sequence coverage of 0.66× generated from multiple pigs [15] has also facilitated the identification of specific genes and should serve as an abundant source of polymorphism data, such as single nucleotide polymorphisms, once a reference sequence is obtained.

For many species, physical maps have played an integral part in the construction of their genome sequence [16-18]. The capacity to select a minimum tile path (MTP) of overlapping clones covering the genome provides not only a substrate for genome sequencing [19,20] but also a resource for the generation of mapped clone arrays for chromosome structure comparison [21,22]. A physical map also has great utility for targeted positional cloning and QTL mapping [23,24]. Porcine genomic resources have been well exploited to provide comprehensive characterization of the genome. Blocks of conserved segments have been established in relation to the human genome. Cytogenetic evidence indicates that the mean lengths of conserved segments are twice as long between the human and pig as between the mouse and human genomes [25,26] and the rate of recombination in the porcine genome has been shown to be lower than in humans [27,28]. These characteristics suggested that the finished human genome would provide a good framework for the construction of a physical clone map of the pig genome through alignment of pig BAC end sequences (BES), using an approach that proved successful in the assembly of the mouse physical map [17].

In this manuscript we address the development of a highly contiguous physical map that integrates radiation hybrid maps and four BAC clone libraries. The BAC map represents the first phase of the international pig genome sequencing project [3]. It provides an immediate resource to the porcine community for rapid electronic positional cloning and a substrate for efficient and economic selection of a MTP of BACs for the determination of the genome sequence. The clone map and BAC end sequence data can also help to support the assembly of maps and genome sequences of other artiodactyls.

## Results

### Fingerprint and BAC end sequence data

The physical map of the pig genome incorporates BAC clones from four different libraries that have been used for positional cloning and to map QTLs in the pig genome. *Hin*dIII restriction digest fingerprints (see Materials and methods) were generated from 264,987 BAC clones, corresponding to approximately 16-fold coverage of the genome (Table 1) based on an estimated genome size of 2.6 to 2.7 Gb [29,30]. The fingerprint coverage comprises 6.7 genome equivalents of the CHORI-242 library [31] generated from a single Duroc sow, approximately four genome equivalents from each of both the PigEBAC (male Large White × Meishan F1) [11] and the RPCI-44 BAC libraries (constructed from pooled material from four male pigs, one-quarter Meishan, three-eighths Yorkshire, and three-eighths Landrace) [32], and 1.5-fold clone coverage of the INRA BAC library (made from a single Large White male) [12].

**Table 1:** Fingerprint and BAC end sequence summary

Library	Fingerprinted clones	Genome complexity	BES passed reads	Paired ends	Average GC %	Average Phred Q20 length (bp)
CHORI-242	101,434	6.7	340,484	93%	41	705
PigEBAC	73,863	4.2	144,870	93%	42	700
RPCI-44	61,225	3.8	71,847	87%	40	521
INRA	28,465	1.5	62,888	94%	42	613
All	264,987	16.2*	620,089	92%	41	635

*Based on a genome size of 2.6 to 2.7 Gb [30].

BES were generated for all clones (see Materials and methods) from the CHORI-242 and PigEBAC libraries, and for fingerprinted clones from the RPCI-44 and INRA libraries (Table 1). The sequences have been deposited in the Ensembl and NCBI trace repositories [33,34] (EMBL:CL320165-CL440255; EMBL:CT033849; EMBL:CT033851-CT476799; EMBL:CR861517-CR925665).

A total of 620,089 high quality sequence reads were produced from 335,463 BACs. After trimming vector sequences, the reads had an average Phred Q20 length of 635 bases and 92% were mated pairs. Taken together, the BES provide a total of 393,756,515 bases representing 15% of the pig genome.

### Porcine BAC end sequence alignment to the human genome: a guide for pig map assembly

We took advantage of the conservation of synteny between the porcine and the human genomes to inform the assembly of the fingerprint map, by aligning the pig BES to Build 35 of the human genome [20] (see Materials and methods). After removal of 19% of BES that contained fewer than 100 bases of continuous, non-repetitive DNA, alignment to the human genome sequence using BLASTN (< 1e^-05^, ≥ 100 bp match, ≥ 66% sequence identity) yielded 297,742 (48% of the total) BES with a match to human sequences (Table 2). The representation of each of the BAC libraries in the aligned set was proportional to the number of clones in the combined BES, indicating that none of the libraries showed any obvious bias (Table 1 in Additional data file 1). Using the requirement that BES alignments to the human genome were consistent with BAC positioning within fingerprint contigs, we established 172,169 high confidence so-called homologous crosslinks [17] between the pig and human genomes (Table 2). The average distance between crosslinks was 17 kb. As observed previously for the mouse genome, the incidence of BES alignments to human chromosomes 19 and Y was lower than observed on other chromosomes. This is most likely due to the large regions of repetitive sequences on these chromosomes that adversely affect the number of unique BES placements. The matching frequency between pig and human genomes is approximately three-fold greater than that observed between mouse and human [17], consistent with the higher level of structural and sequence similarity observed between the pig and human genomes [10,26,35,36].

**Table 2:** Summary of pig BAC end sequence alignments to NCBI build 35 of the human genome

		Human-pig sequence matches		Homologous crosslink matches		Matches to human exons	
				
Human chromosome	Available human sequence (Mb)	Total	Per Mb	Total	Per Mb	Total	Per Mb
1	222.8	24,510	110	14,393	65	3,049	13.7
2	237.5	27,269	115	15,755	66	2,279	9.6
3	194.6	23,691	122	13,991	72	1,949	10.0
4	187.2	20,347	109	11,449	61	1,272	6.8
5	177.7	20,497	115	11,950	67	1,509	8.5
6	167.3	18,898	113	10,856	65	1,586	9.5
7	154.8	15,036	97	8,736	57	1,270	8.2
8	142.6	14,344	101	8,251	58	1,048	7.3
9	117.8	12,597	107	7,286	62	1,092	9.3
10	131.6	13,709	104	8,073	61	1,281	9.7
11	131.1	14,893	114	8,375	64	1,573	12.0
12	130.3	14,817	114	8,427	65	1,778	13.6
13	95.6	9,873	103	5,765	60	628	6.6
14	88.3	10,414	118	6,185	70	1,070	12.1
15	81.3	9,176	113	5,505	68	1,021	12.6
16	78.9	7,129	91	4,233	54	959	12.2
17	77.8	6,949	89	3,969	51	1,365	17.5
18	74.7	8,098	111	5,038	68	521	7.0
19	55.8	2,926	53	1,629	29	1,095	19.6
20	59.5	5,893	99	3,417	57	701	11.8
21	34.2	3,328	98	1,849	54	289	8.5
22	34.8	2,462	71	1,319	38	499	14.3
X	150.4	10,824	72	5,716	38	763	5.0
Y	24.9	62	3	2	0.1	0	0
							
All	2,851.5	297,742	97	172,169	56.3	28,597	10.2

Of the 297,742 BES that were aligned to the human genome, 132,675 (45%) mapped to regions containing annotated genes. Using the 28,597 sequences that overlap at least 1 human exon, it was possible to identify pig BACs that mapped, at least partially, to 11,180 of 21,999 annotated human genes. The distribution of pig BES matches to human exons is shown in Table 2 in Additional data file 1. It is striking that although human chromosome (HAS)19 has the lowest density of pig BES matches, with only 53 per Mb, it has the highest density of exon matches, with 19.6 per Mb. This presumably reflects the fact that HSA19 is the most gene rich of all human chromosomes [37].

The mapping of pig clones to the human genome through BES alignments can be viewed in human Ensembl [38], go to ContigView and turn on Pig BAC ends from the DAS source menu.

### Assembly of the physical BAC map

The assembly of the BAC clone map was affected in two phases. In the first phase the *Hin*dIII digest fingerprint data representing 16 genome equivalents were analyzed and assembled using the FPC database [39]. The initial automated assembly was performed using a cutoff of 10^-10 ^and generated > 12,000 contigs containing binned and ordered overlapping sets of fingerprints. Following preliminary assembly, contigs were merged by identifying fingerprint overlaps using a lower stringency supported by ordering information derived from BES placement on the human genome. In order to avoid 'humanizing' the map assembly, contigs were not joined if the alignment to the human genome was not supported by fingerprint overlap data. In the second phase of the map assembly the consistency of contig joining was assessed and further merges were made. In this phase, human/pig homology breakpoints [36] were targeted using information about the relative positions of markers from the UIUC RH map of the pig genome [10]. The RH map contains 2,413 markers distributed at an average distance of 1 per Mb across the genome and was integrated with the FPC database by fingerprinting 2,083 RPCI-44 clones with BES containing markers positioned on the RH map.

**Figure 1:**
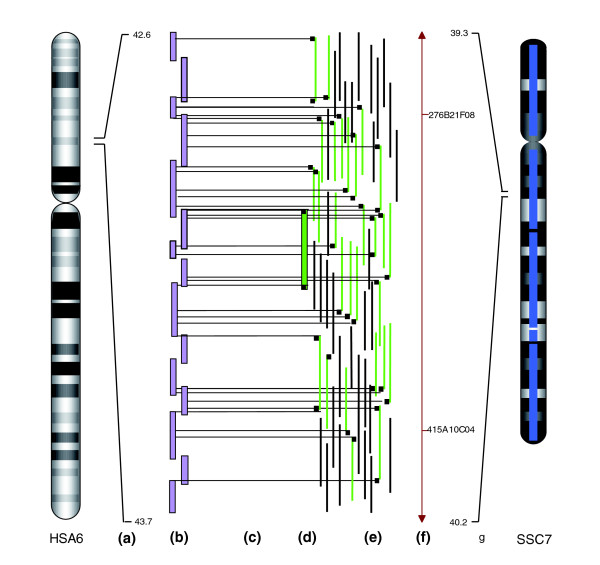
Alignment between human chromosome 6 tilepath and pig chromosome 7 physical map via porcine BES matches. **(a) **Mb scale; **(b) **human sequence tilepath; **(c) **BES matches to human; **(d) **sequenced clone EMBL:CR956379 (CH242-196P11); **(e) **pig clone map - green indicate clones with BAC end sequence match to human; **(f) **UIUC RH map; **(g) **estimated Mb.

**Figure 2:**
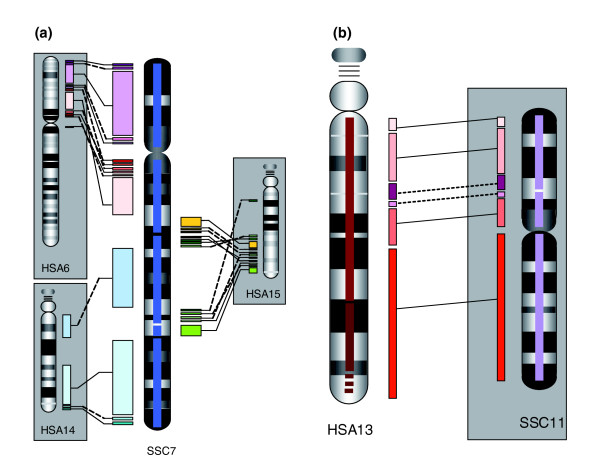
Blocks of conserved synteny between pig and human. **(a) **Pig SSC7 to human chromosomes 6, 14 and 15. **(b) **HSA13 compared to pig chromosome 11. Block inversions between pig and human are denoted with broken lines. Contig coverage is depicted by bars in the center of SSC7 and HSA13.

The incorporation of human-pig homology data combined with fingerprint and RH marker data across a region of HSA6 42.6-43.7 Mb and pig chromosome (SSC)7 39.3-40.2 Mb is illustrated in Figure 1. Using the combined fingerprint, RH map and human/pig conservation of synteny data, we have reduced the number of contigs from 8,718 to 172 with an increase in average contig length from 367 to 15,000 Kb. These data have also been used to construct a detailed map of conserved syntenic blocks between pig and human (Figure 2a, b). The map is accessible through Pre-Ensembl [40].

In the current integrated map, 96% of the RH markers show concordance (2,001/2,083 markers) between their assignment on the RH map and their physical map location, based on positioning of the fingerprint contigs containing BES matches (Figure 1 in Additional data file 1). There are 45 markers that appear to have a different chromosome assignment in the two maps and 37 markers have local mis-ordering on the fingerprint map. We are currently investigating the discrepancies to assess whether the anomalies have arisen as a result of mis-labeling of BES or fingerprints, or whether there are regions of the RH map in which markers are incompletely resolved.

### Genome coverage

The physical map comprises 172 contigs distributed over 18 autosomes and 2 sex chromosomes X and Y (Table 3). Over 98% of the contigs have been placed and ordered on the UIUC RH map and markers designed from BES in the remaining unordered contigs are being mapped on the RH panel to position them in the genome. The level of continuity of the current map is higher than has been achieved for other mammalian genomes using similar approaches, probably reflecting the high level of conserved synteny between the pig and human genomes and the benefit of the availability of the near complete human genome sequence, along with a map of RH markers distributed evenly across the genome. The most contiguous chromosome is the acrocentric SSC13, which shares homology to all of HSA3 and HSA21, and is represented in the current assembly by a single contig of 218 Mb. To our knowledge, this is the longest map contig that has been constructed prior to genome sequencing. Cytogenetic data from the most centromeric and telomeric clones on SSC13 indicate that they coincide with the ends of the chromosome showing good evidence of map completion (Additional data file 2).

**Table 3:** Distribution of contigs across pig chromosomes

	Total		Ordered on UIUC RH map	
		
Pig chromosome	Contigs	Coverage (kb)	Contigs	Coverage (kb)
1	12	300,977	3	295,553
2	15	159,169	8	149,047
3	14	146,851	9	144,675
4	9	143,919	8	143,453
5	8	102,515	4	99,497
6	12	167,617	11	166,045
7	10	136,596	5	132,447
8	5	146,803	4	146,495
9	6	155,323	4	151,750
10	14	80,643	7	75,116
11	4	86,158	3	84,555
12	7	64,461	6	64,189
13	1	218,109	1	218,109
14	4	139,903	3	138,960
15	7	173,355	3	169,770
16	3	86,660	2	86,055
17	6	67,521	5	67,043
18	2	61,577	2	60,404
X	27	127,979	20	125,475
Y	6	1,760	-	-
				
All	172	2,567,896	108	2,518,638

SSCX is currently the most fragmented chromosome, with 27 contigs. There are several possible explanations for the contig number being three- to four-fold higher than might be expected on the basis of size comparison with the autosomes. Firstly, the resolution of the RH map is lower on the X, as it is hindered by the presence of the selectable marker for the panel (HPRT) on this chromosome [8,41]. The clone coverage of SSCX is also slightly lower compared to the autosomes as only one of the BAC libraries, the CHORI-242, is derived from a female animal. Given its homology to other mammalian X chromosomes, it is also likely that continuity of the physical map is affected by the abundance of repetitive elements such as LINE1s [42].

Using the alignment of the map to the human genome sequence and fiber FISH experiments, we estimate that the average size of the map gaps is 200-250 kb, giving an approximate total of 40 Mb not covered in the current map assembly. We are attempting to close the remaining gaps through further screening of the CHORI-242 BAC library and through the construction and end sequencing of a sheared fosmid library generated from DNA used to make the CHORI-242 library.

From karyotyping we have performed there are regions of ribosomal DNA (rDNA) near the centromere of SSC10p and on acrocentric SSC16. Given the repetitive nature of these sequences it is probable that the map lacks complete coverage across these regions.

### Comparison of chromosome size

Our estimates of chromosome size from the fingerprint map are consistent with previous estimates of the relative sizes of the chromosomes, with the exception of chromosomes 14 and 15. It appears from the map that SSC15 (173 Mb) is larger than SSC14 (139 Mb) (Table 3). It is difficult at this stage to determine whether the smaller size estimate of chromosome 14 relative to 15 is due to a higher GC content and gene density reducing the number of *Hin*dIII fragments produced from the BACs and thus resulting in artificial suppression of chromosome size calculated from the fragment number (see Materials and methods). The relative size estimates are consistent, however, with a previous report by Mikawa *et al*. [43]. In addition, the potential presence of un-clonable regions, such as sequences unstable in current BAC vector systems, especially on SSC14, will diminish map coverage. We will aim to cover such regions using alternative vectors, such as fosmids.

### Chromosome Y

Although 50% of the clones fingerprinted came from BAC libraries generated from male pigs, only around 0.25% of BES (1,350) and BAC fingerprints (675) are estimated to derive from the Y chromosome (Table 1). In addition to the small number of sequences, potential BES matches to HSAY may have been excluded due to the repetitive nature of the sequence. However, some coverage across Y has been established. Publicly available gene markers already assigned to clones from the PigEBAC library [44] have been incorporated into the FPC database and have facilitated the detection of contigs for each gene. We anticipate extending the map coverage of the Y chromosome by further integration of Y specific markers or through the incorporation of flow-sorted chromosome Y specific libraries.

### Utility of the physical map for rapid positional cloning of pig genes

**Figure 3:**
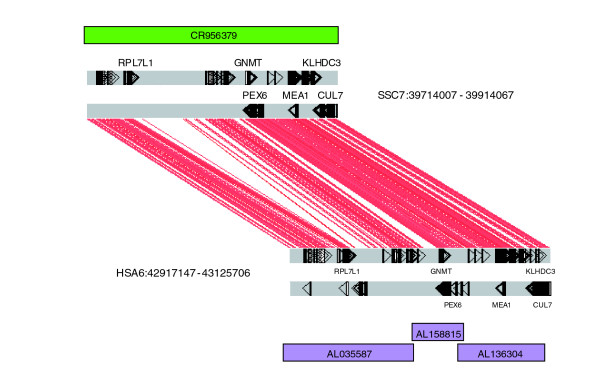
Comparison of homologous annotation between pig and human. Pig clone CH242-196P11 was aligned to HSA6 by BES matches, and the clone was then sequenced [EMBL:CR956379]. The resulting annotation showed the expected homologues to known human genes present in the pig sequence as in human sequences [EMBL:AL035587, EMBL:AL158815, EMBL:AL136304]. Full gene annotation for CH242-196P11 is available in the EMBL entry. Red lines indicate regions of sequence homology with a 75% sequence similarity over the whole region. Figure generated using Artemis Comparison Tool [57].

Positional cloning of regions implicated in disease or traits of agronomic importance has typically involved the identification of regions of interest through large scale quantitative trait locus studies [6], followed by the establishment of a physical map for the region using either homologous coding sequences for the region of interest from human, or localized pig markers to isolate BAC clones experimentally through hydridization or PCR [23]. By systematically generating a whole genome BAC physical map and aligning the BES to the human genome it is now possible to 'electronically clone' regions of importance in the pig genome more rapidly and cost effectively than before. For example, based on the BES alignment between pig and human shown in Figure 1, we were able to identify BAC clone CH242-196P11 spanning 42.9-43.1 Mb on HSA6. Subsequent sequencing and annotation of CH242-196P11 (accession number CR956379) revealed the presence of the same set of genes as found in the homologous region in human (Figure 3).

## Discussion

The publicly accessible integrated physical map described here is being used to select a highly efficient tilepath through the pig genome to provide a substrate for the swine genome sequencing project [3,45]. This follows the strategy used for other large scale mammalian sequencing projects, such as human, mouse and rat [19,20,46]. By using information about the extent of clone overlaps derived from the fingerprint data and re-assessing the relative positions of paired BES alignments to the human genome, we have been able to optimize the selection of an initial tilepath of minimally redundant clones through assembled clone contigs across the pig genome. Subsequent rounds of clone selection will be based on fingerprint data and BES alignments to pig clones. The map may also be improved in an iterative manner as the chromosomes are sequenced and the sequence is used to identify further clones to contiguate the map.

Any collection of clones spanning the genome can act as not only the template for clone based sequencing but also as a resource for functional genomic studies, for example, for gene targeting or to produce mapped BAC arrays. It is our intention to maintain the collection of sequenced clones and to make either individual clones or the complete set available to investigators.

Although a whole genome shotgun strategy for genome sequencing has become very popular and can be used to generate sequence to varying depths for a wide range of animal genomes very rapidly [47,48], physical clone maps continue to provide important orthologous data for the correct assembly of genomes [49]. BAC maps can be used to de-compress regions where highly similar, but non-contiguous repetitive sequences (> 97% identical) are assembled coincidentally [50]. By inspecting BAC contigs demonstrating elevated clone depth (> 3 times expected) and those showing BES alignment to regions of known duplications in the human genome, we have been able to identify approximately 30 regions that are potentially segmentally duplicated in the pig genome. By increasing the map assembly stringency we have begun to resolve these regions prior to tilepath selection and thus improve the coverage of the initial assembly of the pig genome sequence. BAC clones also continue to prove the most effective substrates for finishing genome sequences and thus providing researchers with reference genomes that will have long lasting utility.

## Materials and methods

### Map construction

A bacterial clone physical map of the genome was constructed using *Hin*dIII restriction enzyme fingerprinting [51,52]. BAC library plates were directly cultured into 170 μl 2× TY media in 384-well plates. After overnight growth the BAC DNA was extracted by alkaline lysis on a Packard MiniTrack, and digested with *Hin*dIII in the 384-well plates. Following electrophoresis on 121 lane 1% agarose gels, the data are collected using a Typhoon 8600 fluorimager, raw images were entered into the fingerprint database using the software IMAGE [53]. The output of normalized band values, sizes and gel traces were analyzed in FPC [39], which bins and orders clones on the basis of shared bands, taking marker data into account when available.

The first fingerprint assembly was performed using a cutoff of 10^-10 ^and a fixed match tolerance of 7. The initial set of contigs were merged by ordering them along the human genome through BES matches and, crucially, scrutinizing potential joins by reducing the stringency at which fingerprint overlaps were accepted, where the cutoff was lower than that of the initial build (10^-09 ^to 10^-05^). A further round of merging along each pig chromosome was enabled by incorporation of the UIUC RH map.

### BAC end sequencing

Clones from the CHORI-242 and PigEBAC [8] libraries were end sequenced at the WTSI using the following protocol. BAC clones were inoculated into 1.5 ml of 2× TY media containing 12.5 μg/ml chloramphenicol in 2 ml 96-well growth boxes (Corning Life Sciences, New York, New York, USA) and incubated for 22 hours at 37°C, shaking at 320 rpm. Plates were centrifuged at 4,000 rpm for 3 minutes to obtain pellets, the supernatant was discarded and the cells were re-suspended in100 μl of GTE+RNaseA, and 100 μl of NaOH/SDS was added and mixed before adding 100 μl of 3 M KOAc and further mixing [54]. After filtration through two Millipore plates (MADVN6550/MANUBA50) on a vacuum manifold, the resultant material was dried and re-suspended in 35 μl of 10 mM Tris pH 8.0. The resulting 'prepped' DNA was sequenced with BigDye Terminator Ready Mix v3.0 and BigDye Bufferx5 (Applied Biosystems Foster City, California, USA) using T7 and SP6 primers. The sequenced products were cleaned up by washing with 5 μl 3 M NaOAc and 125 μl 96% ethanol.

The DNA was precipitated by centrifugation at 4,000 rpm for 15 minutes, then washed with a further 100 μl 70% ethanol and the centrifugation repeated. The products were re-suspended in 10 μl of 0.1 M EDTA, pH 7.4 and loaded on to ABI3700 capillary sequencers. Clones from Segment 2 of RPCI-44 porcine BAC library [32] and from Segment 1 of CHORI-242 porcine BAC library were end-sequenced at UIUC using the method described [30]. INRA library BAC clones were end-sequenced at Genoscope (Evry, France) using a method similar to that described above.

### BAC end sequence analysis and placement

BES reads were repeat masked using RepeatMasker (version open-3.0.8, RepBase Update 9.11) [55] and then aligned by BLASTN version 2.0MP-WashU (22-Sep-2003) [56] to NCBI Build35 of the human genome. BLASTN hits (< 1e^-05^, ≥ 100 bp match, ≥ 66% sequence identity) were clustered using the FPC fingerprint assembly. Matches between the pig BES and the human genome had an average score of 866 and an average identity of 74.8%.

### Coverage estimates

The sizes of map contigs were estimated from the number of fingerprint bands using a value of 6.047 kb per band. This value was estimated by dividing the entire sequenced insert of 33 finished pig clones (4,964 Kb) by the number of fingerprint bands detected in each clone (Table 3).

## Additional data files

The following additional data are available with the online version of this paper. Additional data file 1 includes: Table 1, a breakdown of BES matches to human by library; Table 2, listing pig BES matches to human genes; and Figure 1, a plot of RH map (cR) versus physical map (Mb) position for markers on SSC1. Additional data file 2 shows fluorescent *in situ *hybridization (FISH) results for the most centromeric CH242-166N14 (red) and most telomeric clone CH242-248H14 (green) on SSC13.

## Supplementary Material

Additional data file 1 Table 1: breakdown of BES matches to human by library. Table 2: pig BES matches to human genes. Figure 1: plot of RH map (cR) versus physical map (Mb) position for markers on SSC1. Click here for file

Additional data file 2 Fluorescent *in situ *hybridization (FISH) results for the most centromeric CH242-166N14 (red) and most telomeric clone CH242-248H14 (green) on SSC13. Click here for file
