# Supplementary material for: A high utility integrated map of the pig genome
Source: Genome Biol. 2007 Jul 11;8(7):R139. doi: 10.1186/gb-2007-8-7-r139 (PMC2323232; doi:10.1186/gb-2007-8-7-r139)
Supplement: Additional data file 1 — Table 1: breakdown of BES matches to human by library. Table 2: pig BES matches to human genes. Figure 1: plot of RH map (cR) versus physical map (Mb) position for markers on SSC1. [file gb-2007-8-7-r139-S1.doc]

Additional Data Table 1: Breakdown of BES matches to human by library

| Library | Sex of source DNA | BES Generated | | Human – Pig matches | |
| --- | --- | --- | --- | --- | --- |
|  |  | Total | % contribution to total BAC coverage | Total no. of BACs with matches to human | Proportion of total human matches contributed by library |
| CHORI-242 | Female | 340484 | 54 | 161511 | 55 |
| PigEBAC | Male | 144870 | 24 | 72771 | 23 |
| RPCI-44 | Male | 71847 | 11 | 32155 | 12 |
| INRA | Male | 62888 | 11 | 31305 | 10 |
| All |  | 620089 |  | 297742 |  |

Additional Data Table 2: Pig BES Matches to Human Genes

| Human chromosome | Pig BES alignment with human genes | | | |
| --- | --- | --- | --- | --- |
|  | No. of pig BES alignments with entire human exon | No. of human genes matched overlapping entire exon | No. of pig BES alignments with any part of a human gene | No. of human genes with any match |
| 1 | 1803 | 827 | 12382 | 1476 |
| 2 | 1368 | 587 | 11624 | 1003 |
| 3 | 1060 | 498 | 10806 | 880 |
| 4 | 731 | 335 | 7722 | 598 |
| 5 | 836 | 371 | 7991 | 676 |
| 6 | 868 | 395 | 8072 | 742 |
| 7 | 762 | 361 | 7714 | 629 |
| 8 | 584 | 267 | 5472 | 508 |
| 9 | 640 | 296 | 5433 | 529 |
| 10 | 723 | 336 | 6949 | 576 |
| 11 | 881 | 460 | 6513 | 840 |
| 12 | 1060 | 483 | 7150 | 801 |
| 13 | 351 | 130 | 3754 | 273 |
| 14 | 601 | 288 | 4688 | 495 |
| 15 | 609 | 264 | 4526 | 471 |
| 16 | 567 | 307 | 3090 | 530 |
| 17 | 801 | 410 | 4065 | 741 |
| 18 | 300 | 134 | 3158 | 238 |
| 19 | 606 | 360 | 1819 | 660 |
| 20 | 400 | 205 | 2759 | 395 |
| 21 | 165 | 82 | 1204 | 152 |
| 22 | 291 | 162 | 1485 | 291 |
| X | 408 | 228 | 4289 | 478 |
| Y | 0 | 0 | 10 | 5 |
| All | 16415 | 7786 | 132675 | 13987 |

Additional Data Fig 1. Plot of RH map (cR) vs Physical Map (Mb) position for markers on SSC1
