# Supplementary figures and images for: A high utility integrated map of the pig genome
Source: Genome Biol. 2007 Jul 11;8(7):R139. doi: 10.1186/gb-2007-8-7-r139 (PMC2323232; doi:10.1186/gb-2007-8-7-r139)

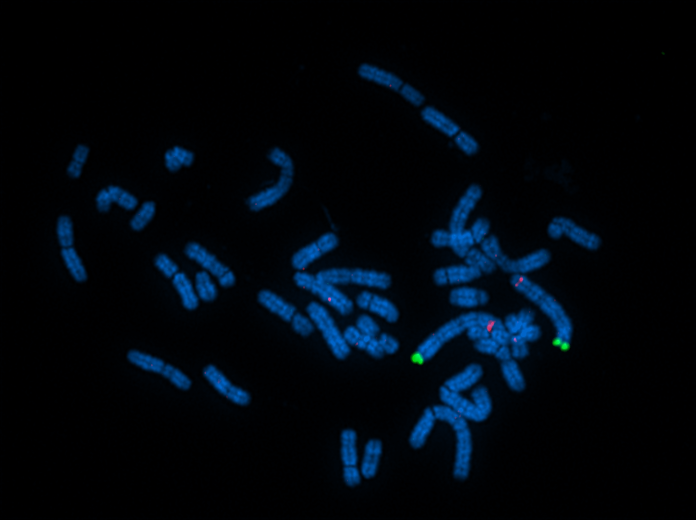

Supplement: Additional data file 2 — Fluorescent in situ hybridization (FISH) results for the most centromeric CH242-166N14 (red) and most telomeric clone CH242-248H14 (green) on SSC13. [file gb-2007-8-7-r139-S2.tiff]
